# Effect of defects on optical and electronic properties of graphene quantum dots: a density functional theory study†

**DOI:** 10.1039/d3ra02564k

**Published:** 2023-05-31

**Authors:** Wei Liu, Yaning Han, Min Liu, Liang Chen, Jing Xu

**Affiliations:** a Department of Optical Engineering, College of Optical, Mechanical and Electrical Engineering, Zhejiang A&F University Hangzhou Zhejiang 311300 P. R. China jingxu@zafu.edu.cn; b School of Physical Science and Technology, Ningbo University Ningbo Zhejiang 315211 P. R. China

## Abstract

The effects of different types of defects (vacancy, Stone–Wales defects, and heteroatom doping) and varying defect concentrations (single and double defects) on the structure, electronic, and optical properties of graphene quantum dots (GQDs) are systematically investigated using time-dependent density functional theory (TD-DFT). The results reveal that most defects induce noticeable structural distortions, with increasing deformation at higher defect concentrations. Compared to pristine GQD model C96 (with a maximum absorption peak at 592 nm), the absorption spectra of 6 defective C96 exhibit blue shifts ranging from 554 to 591 nm, while 12 defective C96 lead to red shifts (598–668 nm). The HOMO–LUMO gaps vary from 0.62 to 2.04 eV (2.10 eV for pristine C96). Quantitative analysis of the absorption spectra and molecular orbital energy levels demonstrate that the electronic and optical properties of defective C96 strongly depend on the types, concentrations, and locations of defects. NTO analysis illustrates that higher electron localization exists in defective C96, which is attributed to the disruption of the original π-conjugation caused by structural distortions and different orbital hybridizations. These findings offer a comprehensive insight into the impact of defects on GQDs and provide valuable guidance for exploiting the unique features of GQDs to expand new applications in various fields.

## Abstract 2

The effects of different types of defects (vacancy, Stone–Wales defects, and heteroatom doping) and varying defect concentrations (single and double defects) on the structure, electronic, and optical properties of graphene quantum dots (GQDs).

## Graphical abstract

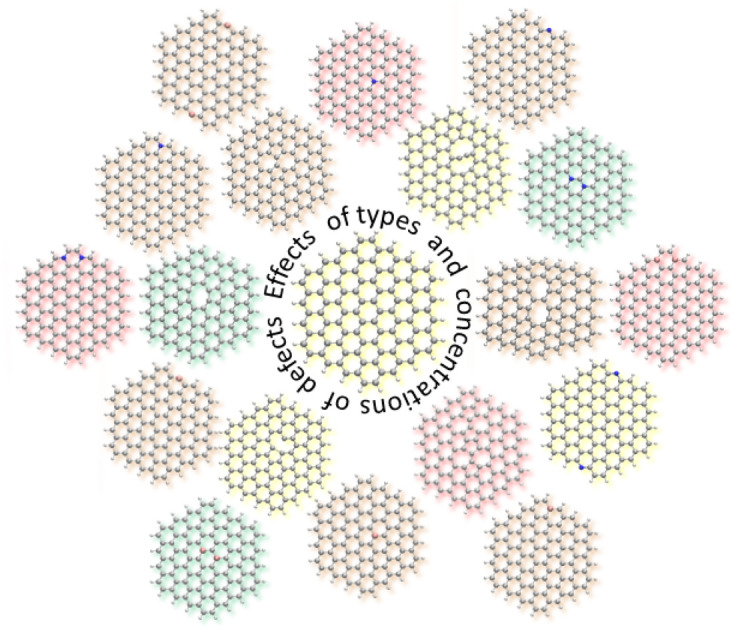

## 1 Introduction

Graphene, a two-dimensional sheet of carbon with a single atomic thickness, has garnered significant attention from the scientific community since its discovery in 2004 due to its exceptional properties, such as high stability, electron mobility, low resistivity, and mechanical flexibility.^1–5^ However, the fact that graphene behaves as a semiconductor with a zero band gap presents a challenge in terms of optical photoluminescence (PL), limiting its potential applications in optoelectronics and photonics.^6–9^ To overcome this limitation, a promising approach is to reduce graphene to zero-dimensions, resulting in the formation of graphene quantum dots (GQDs).^10^ Due to quantum confinement and edge effects, GQDs exhibit unique properties such as adjustable bandgap, high biocompatibility, good photostability and solubility.^11^ These properties make GQDs highly promising for various applications, including sensors, biomedical science, photocatalysis, and energy storage.^12–16^ However, during the preparation process of GQDs, the presence of defects is inevitable and can affect their morphology, structure, and properties.^16–22^ Defects in GQDs can arise from various sources, such as vacancies and topological defects in the top-down process of cutting graphene, or from foreign atom doping in the bottom-up approach using small molecule precursors.^23^ These defects can introduce new electronic states, alter the band gap, affect the optical and electrical properties of GQDs.^24^

The impact of defects on GQDs has been widely investigated, and it has been proven that the type and concentration of defects play crucial roles in determining the properties and applications of GQDs.^16,25–29^ For instance, Basak *et al.* have demonstrated that the introduction of Stone–Wales (SW) defects at specific locations can strongly enhance light absorption in the visible region, making GQDs more valuable for PV applications.^30^ Chakraborti *et al.* have found that an increase in the concentration of SW defects in GQDs results in a decrease in the electrical conductivity of GQDs.^31^ Moreover, some studies have reported that defects induced heteroatom doping, such as nitrogen and sulfur dopants, can reduce the band gap of GQDs, leading to improve light absorption and prolonged luminescence lifetime.^24,32–38^ In addition, the presence of defects in GQDs has been exploited for specific applications. For example, Wang *et al.* develop a carbon-based non-metallic catalyst using defect-rich GQDs, and the catalytic performance of this catalyst depends on the defect density, showing potential for anti-cancer applications.^39^ Ge *et al.* report N/S-doped GQDs, which exhibits multiple state sensitization mechanisms under visible light irradiation, suggesting potential for skin cancer treatment.^40^ Clearly, different types and concentrations of defects can result in different effects and enable new applications. Despite the growing interest in the effects of defects on GQDs, a systematic investigation of the influence of different types and concentrations of defects on GQDs is still desirable, which is crucial for tuning their properties and performance for specific applications.

In this study, we introduce common defects exhibited in the experiment (vacancy defects, SW defects, and heteroatom doping) to the GQD model, and systematically investigate the effect of different types and concentrations of defects on GQDs using time-dependent density functional theory (TD-DFT). The structures, electronic and optical properties are calculated, and absorption spectra and molecular orbital energy levels are quantitatively analyzed. This work provides valuable information that can help us comprehensive understand the absorption mechanism of defective GQDs and guides the regulation of their optical properties by adjusting types and concentrations of defects.

## 2 Methods

All the calculations were performed using Gaussian 16 software.^41^ Geometric optimization, energy and frequency calculations were carried out at the level of B3LYP^42,43^/def2-SVP.^44^ Previous studies have shown that the B3LYP method and def2/SVP basis set have been widely used to explore the structure and electronic properties of graphene and GQD models.^21,45–48^ All obtained local minima have positive frequencies. For the excitation transitions of GQDs, the first 20 lowest excited states were calculated using B3LYP/def2-SVP, and the solvent (water) was included based on the polarizable continuum model (PCM)^49^ in the TD-DFT^29^ calculations. Some computational studies using PCM/TD-DFT have been demonstrated to exhibit good agreement with experimental values.^50,51^ Note that, hydrothermal method is an effective and simple way to prepare GQDs in the experiment,^52–54^ so water is chosen as the solvent in this study. According to the calculation results, molecular orbitals and natural transition orbitals (NTO) were generated using Multiwfn software.^55^

## 3 Results and discussion

**Fig. 1:**
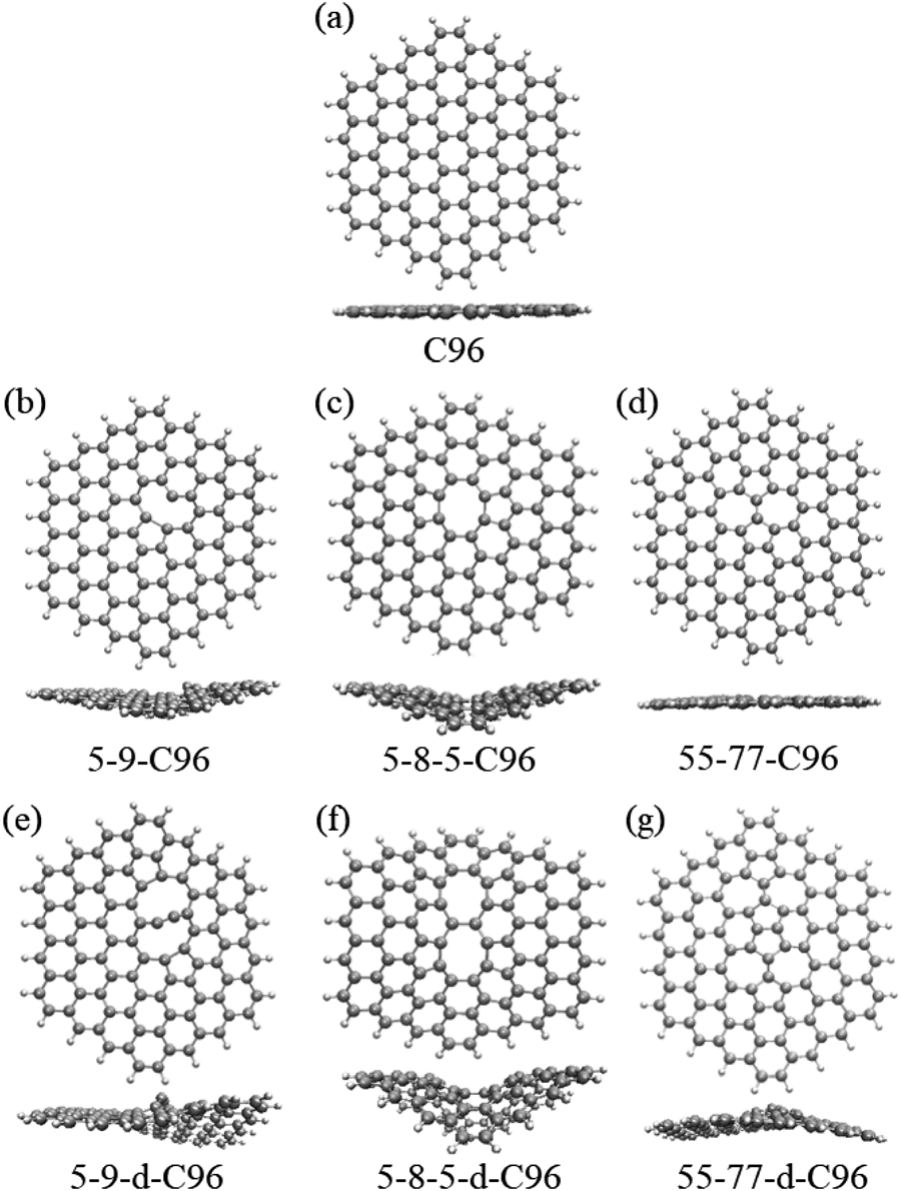
The optimized geometric structures: (a) C96, (b)–(g) Type-I defective C96.

In order to investigate the effects of types and concentrations of defects on GQDs, we utilize C_96_H_24_ hydrocarbon (C96) as a model for pristine GQD (see Fig. 1a) and introduce three common classes of defects in the experiment, including vacancy defects (5-9, 5-8-5), SW defects (55-77), and heteroatom doping (N, B). Two different concentrations of defects, single and double defects, are considered due to the size of the former two classes of defects. As a result, we obtain a total of 18 defective C96, and they are categorized into two types for the convenience of discussion. Type-I are C96 with vacancy defects (5-9, 5-8-5) and SW defects (55-77), which are formed by carbon atom vacancy and C=C bond rotation, respectively. In this type of defect, the single and double defect systems are named X-C96 and X-d-C96 (X represents 5-9, 5-8-5, or 55-77), respectively. Type-II are C96 with externally doped heteroatoms X (B or N atoms), where X replaces C atom on the edge, C–H group on the edge, or C atom on the surface. According to the location and concentration of heteroatom substitution, Type-II are further divided into three sub-types, *i.e.*, Type-II-a (X-edge-C96 and X-edge-d-C96), Type-II-b (X-pyr-C96 and X-pyr-d-C96), and Type-II-c (X-surf-C96 and X-surf-d-C96).

**Fig. 2:**
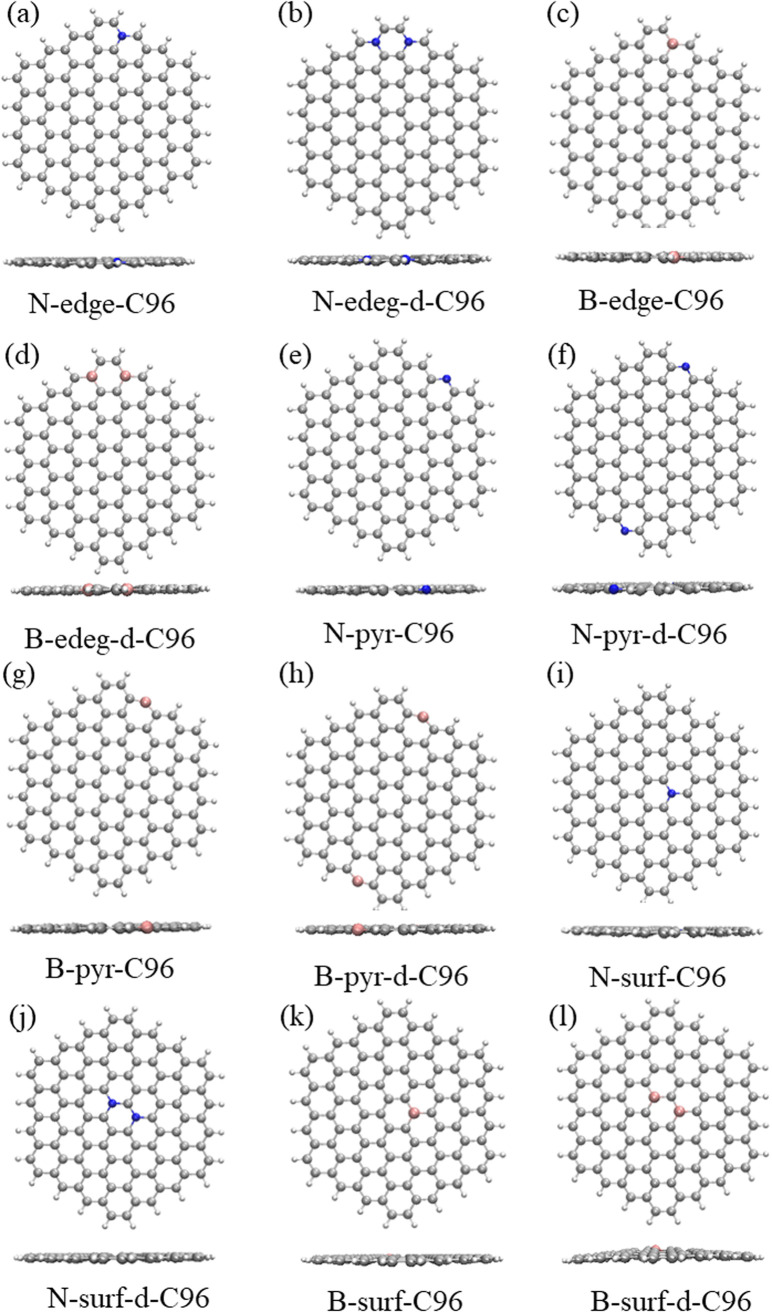
The optimized geometric structures: (a)–(d) Type-II-a defective C96, (e)–(h) Type-II-b defective C96, (i)–(l) Type-II-c defective C96.

Fig. 1 and 2 show the optimized geometric structures of C96 and 18 defective C96 at the level of B3LYP/def2-SVP level. For Type-I, the presence of defects can cause geometric distortions in structures with single defects compared to the pristine model (see Fig. 1a–d). Among them, 55-77-C96 exhibits the least structural change, with only one C=C bond rotates, while other two systems form larger octagonal ring or nine-membered ring due to the loss of one or two C atoms, resulting in significant geometric deformations. We observe that the extent of geometric distortion increased with an increase in defect concentration (see Fig. 1e–g). This phenomenon can be attributed to the formation of more non-hexagonal rings, introducing Gaussian curvature and causing substantial geometric deformations. As shown in Fig. 2, in contrast to Type-I structures, Type-II structures exhibit very weak geometric deformations. However, the degree of geometric deformation still varies slightly, depending on the doping location and the type of doping atom. For example, surface doping has a greater effect than edge doping, and the effect is more pronounced when the doping atom is B. Overall, the effect of external heteroatoms on the skeleton is very weak.

**Fig. 3:**
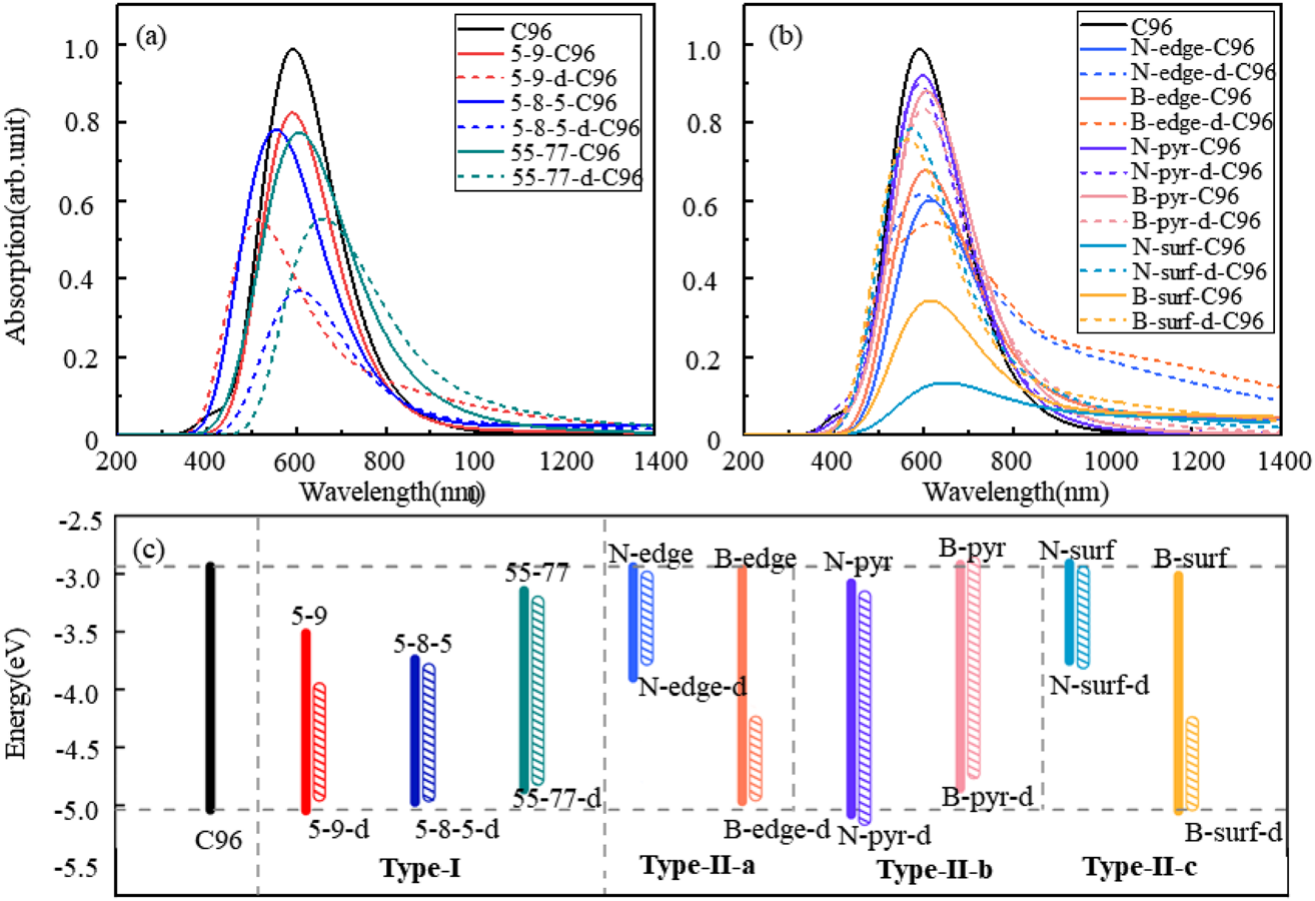
The calculated absorption spectra of C96 and 18 defective C96 systems: (a) Type-I, (b) Type-II, (c) the HOMO–LUMO gaps of pristine and defective C96 systems.

The calculated absorption spectra of C96 and 18 defective C96 in water are presented Fig. 3a–b. The calculated absorption maximum (*λ*_max_) of C96 is found to be 592 nm. In comparison with C96, Type-I single-defect C96 exhibit a slight decrease in absorption intensity, and the absorption maxima *λ*_max_ vary depending on the type of defects. Notably, 5-9-C96 (*λ*_max_ = 591 nm) shows minimal change in absorption compared to C96, while 55-77-C96 (*λ*_max_ = 606 nm) exhibits a slight red shift. Specific analysis will be discussed later in combination with the molecular orbital and the HOMO–LUMO gap. On the other hand, 5-8-5-C96 is significantly affected, showing a substantial blue shift with a maximum absorption peak at 554 nm. As the defect concentration increases, C96 with double defects also display a significant decrease in absorption intensity, with 5-8-5-d-C96 exhibiting the most obvious decrease. The maximum absorption peak of 5-9-d-C96 continues to show a slight blue shift, while 5-8-5-d-C96 and 55-77-d-C96 both exhibit red shifts compared to single defects. For Type-II defective C96, the diversity of doping types complicates the situation. Firstly, for edge-doped cases, Type-II-a defective C96 result in a red shift in their absorption spectra, which becomes more pronounced with increasing dopant concentrations. In contrast, Type-II-b defective C96, including single and double doping, show only slight changes in their absorption peak positions and intensities, where only N-pyr-d-C96 shows a blue shift and the rest systems exhibit a red shift. This is consistent with previous study that double N-doping is found to cause slight blue shift, while double B-doping can cause slight red shift.^21^ In the case of surface-doped Type-II-c C96, the absorption peaks of single-atom-doped C96 show a significant red shift and the absorption intensity decreases obviously, while the absorption spectra of double-atoms-doped C96 exhibit a significant blue shift, with an increase in absorption intensity compared to single-atom-doped C96 systems. In general, in Type-II systems, surface doping has a greater impact on the absorption intensity compared to edge doping. Additionally, B doping has a greater influence on the absorption spectra compared to N doping except N-surf-C96. The above results show that the absorption spectra of C96 in Type-I and Type-II systems are significantly affected by the defect type, defect concentration and geometric deformation caused by defects.

To gain a more comprehensive understanding of the influence of these defects on the optical properties of C96, we calculate the energy levels of the highest occupied molecular orbital (HOMO) and lowest unoccupied molecular orbital (LUMO) for both pristine and defective C96 systems, which usually determine the absorption spectra of GQDs. The values of all the HOMO–LUMO gaps are listed in Table S1,† and the corresponding diagram is shown in Fig. 3c. The HOMO–LUMO gap of pristine C96 is 2.10 eV, and those of 18 defective C96 range from 0.62 to 2.04 eV. Clearly, the type and concentration of defects can directly impact the energy levels of GQD model. As shown in Fig. 3c, the HOMO–LUMO gaps of Type-I single-defect GQD models are 1.60 eV, 1.25 eV, and 1.74 eV, respectively, which are smaller than the value of pristine GQD model. With the increase of defect concentration, the gap reduction increases slightly. Similar to absorption spectra, the energy level changes for Type-II are also complex. For Type-II-a and Type-II-c, when the doping atom is N, the HOMO–LUMO gaps significantly decrease (0.96 and 0.84 eV), while when the doping atom is B, only a weak change occurs. As the doping concentration increases, the gaps decrease obviously compared to the pristine GQD. For Type-II-b, the influence of doping atoms on gaps is very small, which is consistent with the results of the spectral analysis mentioned above.

**Fig. 4:**
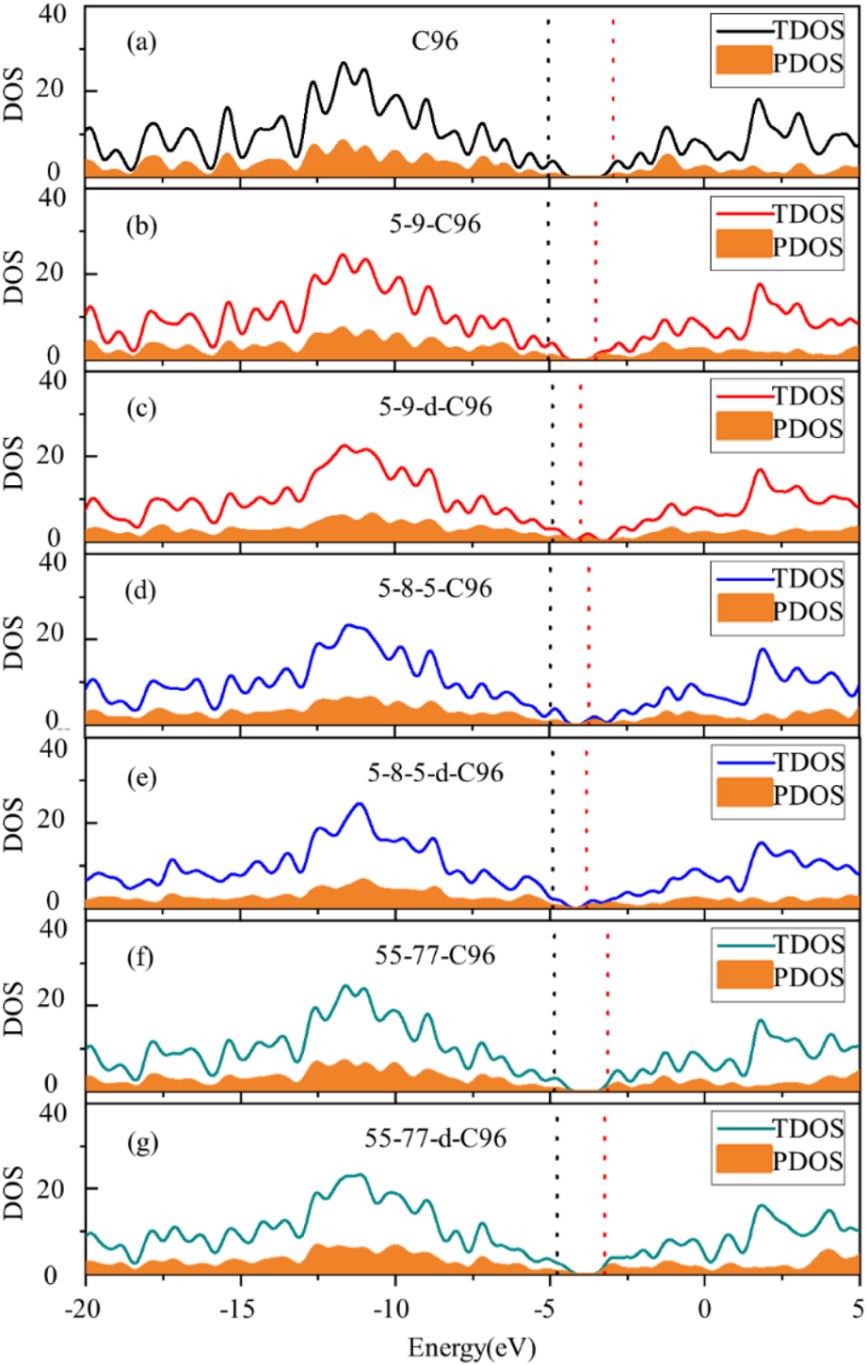
Density of states (DOS) of (a) C96 and (b)–(g) Type-I defective C96 systems. The solid line represents the TDOS. The yellow shaded regions represent the PDOS for the 10 benzene rings in C96, as well as the rings containing all defects in defective C96 models (The black dashed line represents the position of HOMO, the red dashed line represents the position of LUMO, and the DOS is plotted with an electron volt width of 0.5 eV.).

In order to more deeply elucidate the influence of defect type and concentration on the orbital energy level changes mentioned above, we further perform an electronic structure analysis by calculating the total density of states (TDOS) and partial density of states (PDOS) for both pristine and defective GQD models. The TDOS curves are derived from the distribution of molecular orbital energy levels, and the TDOS and PDOS plots visually demonstrate the molecular orbital compositions and contributions of fragments in GQDs. Here, we take Type-I defective GQDs as the example to discuss in detail and show TDOS and PDOS plots of pristine and Type-I defective GQD models in Fig. 4. The plots for other systems can be found in ESI Fig. S1–S3.† As shown in Fig. 4a, the PDOS curve of the 10 benzene rings in pristine C96 is similar to the TDOS curve, indicating that the hexagonal carbon ring dominates the electronic structure of pristine GQD model. However, the DOS change in defective GQD models vary with the type and concentration of defects, suggesting that electronic transitions can be affected by defect type and defect concentration. For example, the HOMO energy levels of all Type-I defects remain almost unchanged, but their LUMO energy levels shift to the left. Among them, the TDOS and PDOS of 5-9-C96, 5-9-d-C96, 5-8-5-C96, and 5-8-5-d-C96 almost overlap near LUMO. This indicates that these four classes of vacancies defects all have the great contributions to LUMO, resulting in the great degree of left shift of LUMO. The contributions of 55-77 and 55-77-d defects to LUMO are relatively small, so their LUMO changes are not significant. The differential contributions of these defects to the HOMO and LUMO energy levels account for the observed changes in the band gap, as analyzed previously.

**Fig. 5:**
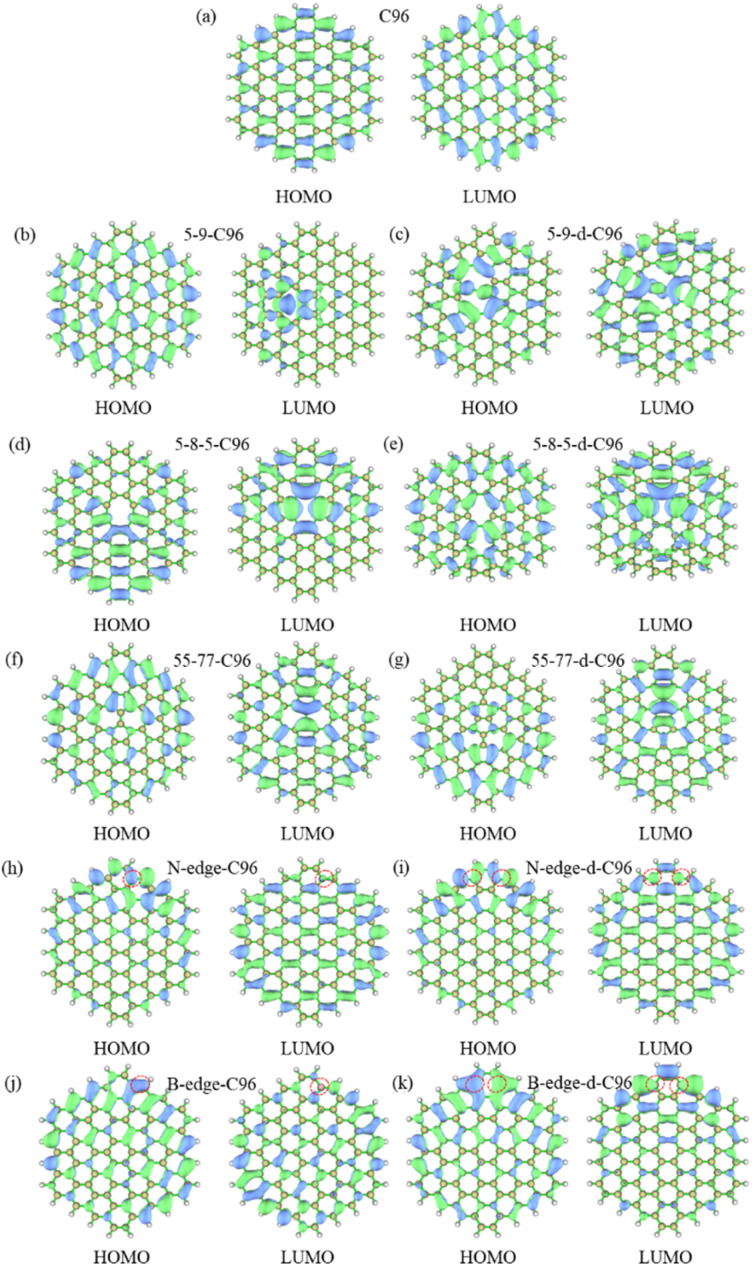
The isosurfaces of HOMO and LUMO for (a) C96, (b)–(g) Type-I defective GQD models, (h)–(k) Type-II-a defective GQD models.

The frontier orbital hybridization is also an important factor that affects the properties of GQDs. Therefore, the isosurfaces of HOMO and LUMO shown in Fig. 5 and S4† are also plotted in this study to explore the effect of defects on the frontier orbital hybridization of GQDs. The delocalized π bonds usually play a significant role in their optical properties of GQDs. In pristine C96, as shown in Fig. 5a, the delocalized π bonds are uniformly distributed on the carbon skeleton of both HOMO and LUMO. The molecular orbital of C96 is consistent with the result reported previously, where the HOMO orbital has e_2u_ symmetry, and the LUMO orbital has e_1g_ symmetry, resulting in the aromaticity pattern of C96 is Clar-type.^56^ For Type-I defective C96, the HOMO of 5-9-C96 is similar to that of pristine GQD model, with uniformly distributed π bonds throughout the molecule surface, however, the π bonds of LUMO orbitals mainly localize around the defects (see Fig. 5b). This is consistent with the previous analysis of energy level changes, that is, the 5-9 defect has a weak effect on HOMO energy level of C96, but a significant effect on the LUMO energy level. For GQD models with 5-8-5, 55-77 defects shown in Fig. 5d–g, their π bonds of HOMO and LUMO are mainly concentrated around the defects. This indicates that these two defects weaken the integrity of the π conjugated system. In all the Type-I defective GQD models, with the increase of defect concentration, stronger electron localization can be observed, which leads to greater changes in HOMO and LUMO levels, especially for 5-9-d-C96. From Fig. 5h–k, the HOMO and LUMO of Type-II-a defective GQD models also exhibit disrupted π conjugated systems, mainly distributed around the doped N/B atoms. Doping B atoms in HOMO can result in delocalized electrons due to the formation of 3-center 2-electron bonds. Furthermore, we find that the localization of HOMO and LUMO in doped GQDs increases with increasing doping concentration. This may be attributed to the fact that doping two N/B atoms under the same benzene ring leads to more structural distortions and hybridized orbitals, and this larger orbital hybridization is also responsible for the smaller HOMO–LUMO gaps observed in double-atoms-doped GQD models, as described in the previous analysis. Type-II-c defective GQD models (Fig. S4†) have similar conclusions to Type-II-a, but the situation in Type-II-b defective GQD models are much different. In Type-II-b, the distributions of π bonds in single-atom-doped C96 systems are similar to that in double-atoms-doped C96 systems, indicating similar hybrid orbitals for both doping concentrations. This may be due to the fact that the two doping atoms (N/B) are doped on different benzene rings, resulting in similar hybrid orbitals for both concentrations of doping. In all, our research results demonstrate that different types, concentrations and positions of defects can lead to different orbital rearrangements, further impacting on the electronic and optical properties of GQD models.

**Fig. 6:**
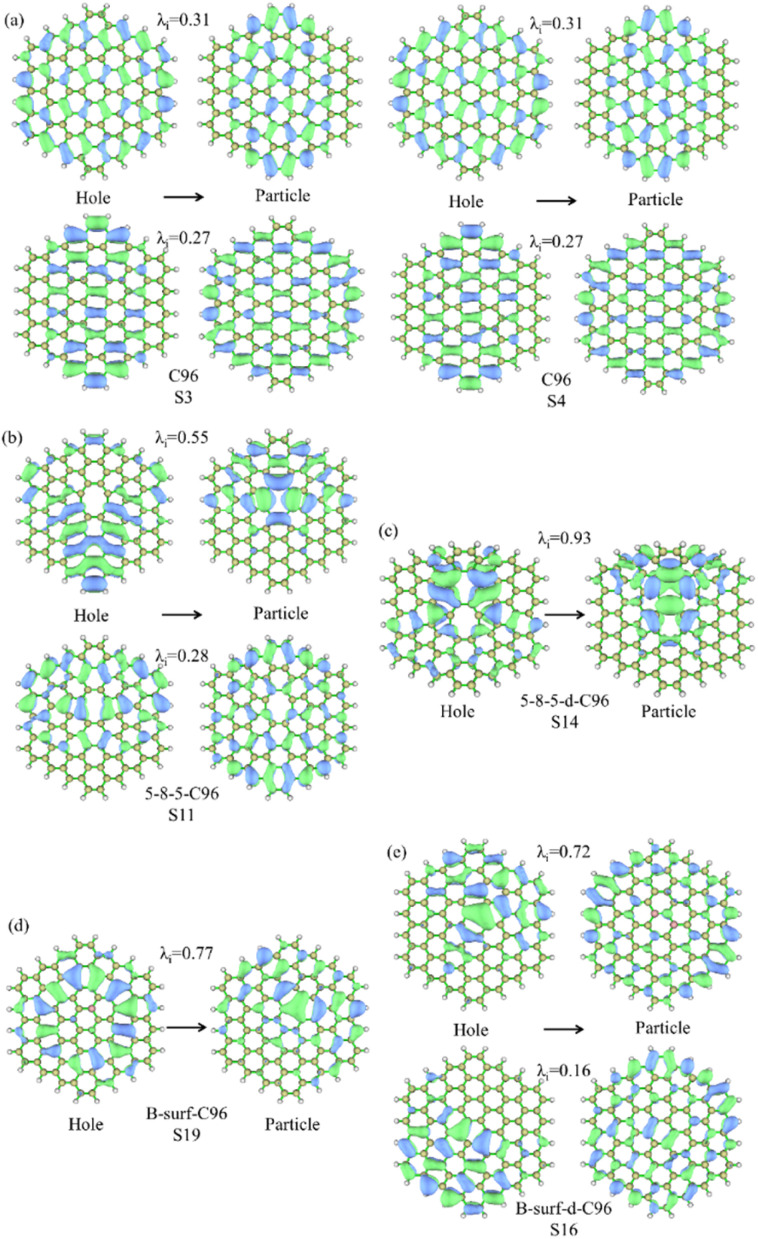
NTO analysis for the prominent excited states of (a) C96, (b) 5-8-5-C96, (c) 5-8-5-d-C96, (d) B-surf-C96 and (e) B-surf-d-C96. The hole is on the left and the particle is on the right; *λ*_i_ represents the corresponding eigenvalue.

For the further investigation of the PL of GQDs, we performed TD-DFT calculations on the first 20 lowest excitations of pristine and defective GQD models. Table 1 lists the main contributing excited states, excitation energies, wavelengths, oscillator strengths, and orbital transition coefficients for all systems. C96 has two similar dominant excitations, S3 and S4, with the same excitation energy of 2.09 eV. S3 mainly consists of the combined transitions of H-1 → L+1 (0.48) and H → L (0.48), and S4 excitation is a mixed transition between H-1 → L (0.48) and H → L+1 (0.48), where H and L represent the HOMO and LUMO orbitals, and the numbers in parentheses denote the orbital transition coefficients. It can be seen that the dominant excited state in pristine GQD is not described solely by a single transition orbital, and similar situations are also observed in defective GQDs. For example, the main contribution of 5-9-C96 is S8, which is a mixed transition between H-1 → L+2 (0.46) and H → L+1 (0.43). In addition, the orbital transition modes of defective C96 systems are varied, and it is difficult for some systems to clearly identify the major transition contributions and accurately describe the transition process. Natural Transition Orbitals (NTOs) are a transformation mode of the canonical orbitals, which can decompose a specific excited state transition into one or two pairs of orbitals. Each orbital is paired with an unoccupied orbital and weighted with appropriate characteristic values to facilitate orbital analysis.^57^ Therefore, we perform corresponding NTO analysis on significant transitions for pristine and defective GQD models using the TD-DFT method and obtain the corresponding associated eigenvalues. Here, we take C96, 5-8-5-C96, 5-8-5-d-C96 in Type-I, and B-surf-C96 and B-surf-d-C96 in Type-II as representative examples. Their NTO diagrams and corresponding associated eigenvalues are displayed in Fig. 6, and the corresponding results of the remaining systems are shown in Fig. S5.† From Fig. 6, two pairs of NTOs describe the degree of electron delocalization in C96 showing S0 → S3 and S0 → S4, where the electron clouds of S3 and S4 transitions exhibit similar delocalization. Defective C96 systems can be described using one and two pairs of NTOs, respectively. Compared to C96, the hole and the particle of defective GQD models undergo certain changes, and the electron distributions are more localized. The wave functions of NTO in 5-8-5-d-C96 are mainly localized in certain regions, which may be because the double defects cause more obvious structural distortion of C96 (as shown in Fig. 1). For C96 systems doped with heteroatoms, although the radius of doped atom is similar to that of C and does not cause significant structural deformation, the presence of external atoms can cause different orbital hybridization and changes in electron cloud distribution. For example, the hole and particle of B-doped C96 systems also exhibit high localization due to the electron-deficient nature of B atom and their propensity to form 3-center 2-electron bonds. Based on these results, the optical properties of GQDs can be effectively regulated by adjusting the type and concentration of defects.

**Table 1:** The excitation energy, wavelength, oscillator strength, and transition coefficient of the main excited states in pristine C96 and 18 defective GQDs. Ha and Hb represent the alpha and beta orbitals, respectively

GQDs	Dominant excitation	Excitation energy (eV)	Wavelength (nm)	Oscillator strength (f)	Transition coefficients	
C96	S3	2.09	592	1.71	H-1 → L+1	0.48
					H → L	0.48
	S4	2.09	592	1.71	H-1 → L	0.48
					H → L+1	0.48
5-9-C96	S8	2.10	591	1.42	H-1 → L+2	0.46
					H → L+1	0.43
5-9-d-C96	S18	2.41	515	0.71	H → L+4	0.41
					H → L+3	0.26
5-8-5-C96	S11	2.24	554	0.77	H-6 → L	0.60
					H → L+2	0.15
					H-1 → L+1	0.12
5-8-5-d-C96	S14	2.04	606	0.62	H-3 → L+1	0.41
					H → L+2	0.27
55-77-C96	S7	2.04	606	1.12	H-1 → L+1	0.80
					H → L+2	0.10
55-77-d-C96	S8	1.88	659	0.71	H → L+2	0.52
					H-1 → L+1	0.25
N-edge-C96	S16	2.03	611	0.76	Ha → La+6	0.21
					Ha-1 → La	0.16
N-edge-d-C96	S10	1.90	652	0.82	H-1 → L	0.73
					H → L+6	0.14
B-edge-C96	S14	2.00	619	0.48	Hb−7 → Lb	0.15
					Ha-1 → La	0.13
					Hb → Lb+2	0.12
B-edge-d-C96	S8	1.86	668	0.81	H → L+1	0.87
N-pyr-C96	S3	2.07	598	1.52	H → L+1	0.46
					H-1 → L	0.40
N-pyr-d-C96	S3	2.10	591	1.61	H-1 → L	0.52
					H → L+1	0.45
B-pyr-C96	S3	2.04	607	1.34	H-1 → L	0.58
					H → L+1	0.31
B-pyr-d-C96	S6	2.06	601	1.59	H-1 → L	0.59
					H → L+1	0.37
N-surf-C96	S18	1.91	650	0.16	Ha−1 → La+1	0.38
					Ha-2 → La	0.28
N-surf-d-C96	S16	2.16	572	1.10	H-2 → L+1	0.52
					H-1 → L	0.21
					H-1 → L+2	0.18
B-surf-C96	S19	2.01	615	0.62	Ha → La+1	0.28
					Hb → Lb+1	0.28
					Ha-2 → La	0.20
B-surf-d-C96	S16	2.19	565	0.99	H-1 → L+2	0.50
					H-10 → L	0.23

## 4 Prospects

In this study, we present a systematic investigation on the impact of defects on the C_96_H_24_ model. Previous research has demonstrated that the structure, the molecular orbital symmetry and the aromaticity pattern of polycyclic aromatic hydrocarbons with a general formula of C_6*n*_^2^H_6*n*_ depend on the parity of *n*.^56^ C_96_H_24_ used as the model in this paper is an even *n* system (*n* = 4), where its HOMO orbital has e_2u_ symmetry and LUMO orbital has e_1g_ symmetry, resulting that its aromaticity pattern is Clar-type. To further explore the effects of defects, we expand our analysis to an odd *n* system. We choose C_150_H_30_ (C150) as the model with *n* = 5. The HOMO and LUMO orbital of C150 exhibit e_1g_ and e_2u_ symmetries, respectively, and its aromaticity pattern is non-Clar. Here, all Type-I defects are introduced into C150 as the examples, and present the optimized geometric structures, the calculated absorption spectra, HOMO–LUMO gaps, and isosurfaces of HOMO and LUMO in Fig. S6–S8.† Similar to the even *n* case, the geometric deformations are also observed in all the defective C150, with 55-77 defect having the weakest influence. Our calculations reveal that *λ*_max_ of C150 model is 767 nm. Upon introducing defects, the absorption intensities decrease, and the maximum absorption peaks show blue shifts. The degree of these changes increases with the higher defect concentrations. Analysis of the molecular orbitals shows that π bonds are evenly distributed in both HOMO and LUMO levels of C150. However, after introducing defects, the π bond distribution in the HOMO of defective C150 remains similar to that of C150, while π bonds in the LUMO mainly localize around the defects. As a result, the defects have a smaller impact on the HOMO energy level but a larger impact on the LUMO energy level, ultimately reducing the HOMO–LUMO gaps mainly due to the LUMO energy level changes. Clearly, for models with odd *n*, the impact of defect types and concentrations is also complex and variable. Previous studies have indicated that the electronic and optical properties of GQD models are influenced by factors such as substrate size, edge morphology, and shape.^58^ Hence, it is important and worthwhile to continue systematically exploring the influence of defect types and concentrations on various GQD models at the microscopic level in the future. This endeavor will provide valuable and comprehensive insights, enhancing our understanding of GQDs with defects.

## 5 Conclusions

In conclusion, we systematically investigate the effect of defect type and concentration on the structures, electronic, and optical properties of C96 model using the TD-DFT approach. The computational results show that C96 systems with defects exhibit structural distortions, where systems with non-hexagonal rings have the greatest influence. Quantitative analysis of the absorption spectra and molecular orbital energy levels has revealed that the changes of electronic and optical properties of defective C96 strongly depend on the type, concentration, and location of defects. NTO analysis has shown that structural distortions and different orbital hybridizations disrupt the original π-conjugation system of C96. Additionally, a extend model C150 with odd *n* are also consistent that the impact of defect types and concentrations is complex and variable. This work provides valuable insights into the absorption mechanism of C96 model with different defect types and concentrations, and the potential for tuning GQD optical and electronic properties based on defects for potential applications.

## Author contributions

W. L., Y. H. and J. X. conceived the research; Y. H. and W. L. performed the calculations and analyzed the data; W. L., Y. H. and J. X. wrote the manuscript; M. L. and L. C. helped to revise the manuscript. All authors discussed and commented on the manuscript.

## Conflicts of interest

There are no conflicts to declare.

## Supplementary Material

RA-013-D3RA02564K-s001
