# Supplementary material for: Effect of defects on optical and electronic properties of graphene quantum dots: a density functional theory study
Source: RSC Adv. 2023 May 31;13(24):16232–40. doi: 10.1039/d3ra02564k (PMC10230513; doi:10.1039/d3ra02564k)
Supplement: RA-013-D3RA02564K-s001 [file RA-013-D3RA02564K-s001.pdf]

Electronic Supplementary Material (ESI) for RSC Advances.
This journal is © The Royal Society of Chemistry 2023

Supplementary Information

Effect of Defects on Optical and Electronic Properties of Graphene Quantum Dots: A Density Functional Theory Study

Wei Liu[1,†], Yaning Han[1,†], Min Liu[1], Liang Chen[2,1], and Jing Xu[1,]*

[1] *Department of Optical Engineering, College of Optical, Mechanical and Electrical Engineering, Zhejiang A&F University, Hangzhou, Zhejiang, 311300, P. R. China*

[2] *School of Physical Science and Technology, Ningbo University, Ningbo, Zhejiang, 315211, P. R. China*

[†] *Wei Liu and Yaning Han contributed equally to this work.*

Email: jingxu@zafu.edu.cn

Contents

Fig. S1 DOS of C96 and Type-II-a defective C96……………….…………………………..….......**3**
Fig. S2 DOS of C96 and Type-II-b defective C96……………….………………………….....…...**4**
Fig. S3 DOS of C96 and Type-II-c defective C96……………………………………………….....**5**
Fig. S4 HOMO and LUMO of Type-II-b defective C96 and Type-II-c defective C96....……….…**6**
Fig. S5 NTO analysis for the prominent excited states of Type-I defective C96 and Type-II defective C96………....……………………...………………………………………………………...**7**
Fig. S6 The geometric structures of the optimized C150 and Type-I defective C150…………..……..**10**
Fig. S7 The calculated absorption spectra and HOMO-LUMO gap of C150 and Type-I defective C150………..……………………………………………………………………………....**10**
Fig. S8 The isosurfaces of HOMO and LUMO for C150 and Type-I defective C150……………**11**
Table S1 The HOMO energies, LUMO energies and HOMO-LUMO gaps for C96 and defective C96………...……………………………………………………………………………**12**
Table S2 Absorption energies, wavelengths, and oscillator strengths for the first 20 singlet excited states of C96………... …………………………………………………………………..……**13**
Table S3 Absorption energies, wavelengths, and oscillator strengths for the first 20 singlet excited states of 5-9-C96. ……………………………………………………………………..………**14**
Table S4 Absorption energies, wavelengths, and oscillator strengths for the first 20 singlet excited states of 5-9-d-C96…………………...…………………………………………………….…**15**
Table S5 Absorption energies, wavelengths, and oscillator strengths for the first 20 singlet excited states of 5-8-5-C96. ……………………………………………………………………..………**16**
Table S6 Absorption energies, wavelengths, and oscillator strengths for the first 20 singlet excited states of 5-8-5-d-C96. ……………………………………………………………….……….**17**

Table S7 Absorption energies, wavelengths, and oscillator strengths for the first 20 singlet excited states of 55-77-C96. ………………...…………………………………………………………..**18**
Table S8 Absorption energies, wavelengths, and oscillator strengths for the first 20 singlet excited states of 55-77-d-C96. ………………..………………………………………………………..……**19**
Table S9 Absorption energies, wavelengths, and oscillator strengths for the first 20 singlet excited states of N-edge-C96. ………………..…………………………………………………………**20**
Table S10 Absorption energies, wavelengths, and oscillator strengths for the first 20 singlet excited states of N-edge-d-C96. . ………………..………………………………………………………....…**21**
Table S11 Absorption energies, wavelengths, and oscillator strengths for the first 20 singlet excited states of B-edge-C96…..………………..…………………………………………………………..…**22**
Table S12 Absorption energies, wavelengths, and oscillator strengths for the first 20 singlet excited states of B-edge-d-C96. ……………..…………………………………………..……….…….**23**
Table S13 Absorption energies, wavelengths, and oscillator strengths for the first 20 singlet excited states of N-pyr-C96…...……………..…………………………………………..…………..…**24**
Table S14 Absorption energies, wavelengths, and oscillator strengths for the first 20 singlet excited states of N-pyr-d-C96……………..…………………………………………..……….…….**25**
Table S15 Absorption energies, wavelengths, and oscillator strengths for the first 20 singlet excited states of B-pyr-C96…...……………..…………………………………………..………..……**26**
Table S16 Absorption energies, wavelengths, and oscillator strengths for the first 20 singlet excited states of B-pyr-d-C96……………..…………………………………………..……….…….**27**
Table S17 Absorption energies, wavelengths, and oscillator strengths for the first 20 singlet excited states of N-surf-C96…………...….…………………………………………..…………..…**28**
Table S18 Absorption energies, wavelengths, and oscillator strengths for the first 20 singlet excited states of N-surf-d-C96…………...….…………………………………………..…………..…**29**
Table S19 Absorption energies, wavelengths, and oscillator strengths for the first 20 singlet excited states of B-surf-C96…………...….…………………………………………..…………..…**30**
Table S20 Absorption energies, wavelengths, and oscillator strengths for the first 20 singlet excited states of B-surf-d-C96. ………………..…………………………………………..…………....…**31**

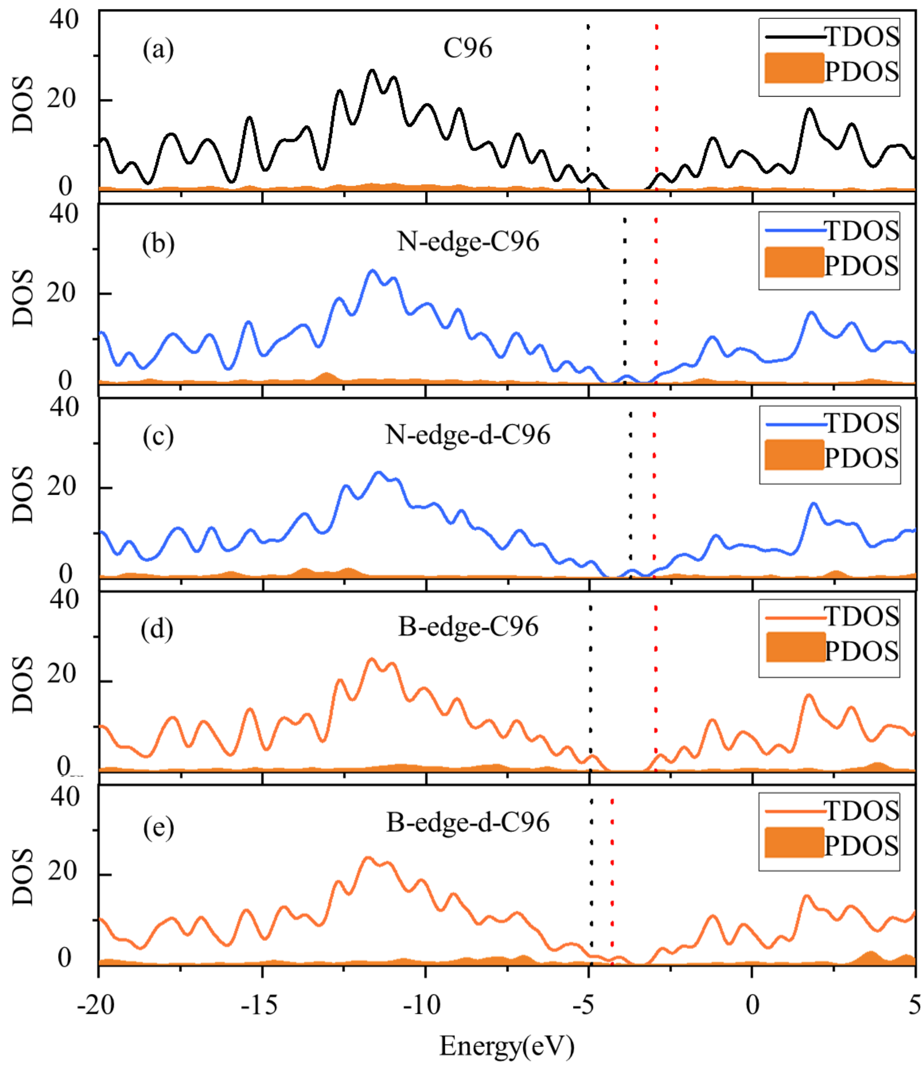

Fig. S1 DOS of C96 and Type-II-a defective C96. The solid line represents the TDOS. The yellow shaded regions represent the PDOS for one benzene ring in C96, as well as the rings containing all defects in the defective C96. (The black dashed line represents the position of HOMO, the red dashed line represents the position of LUMO, and the DOS is plotted with an electron volt width of 0.5 eV.)

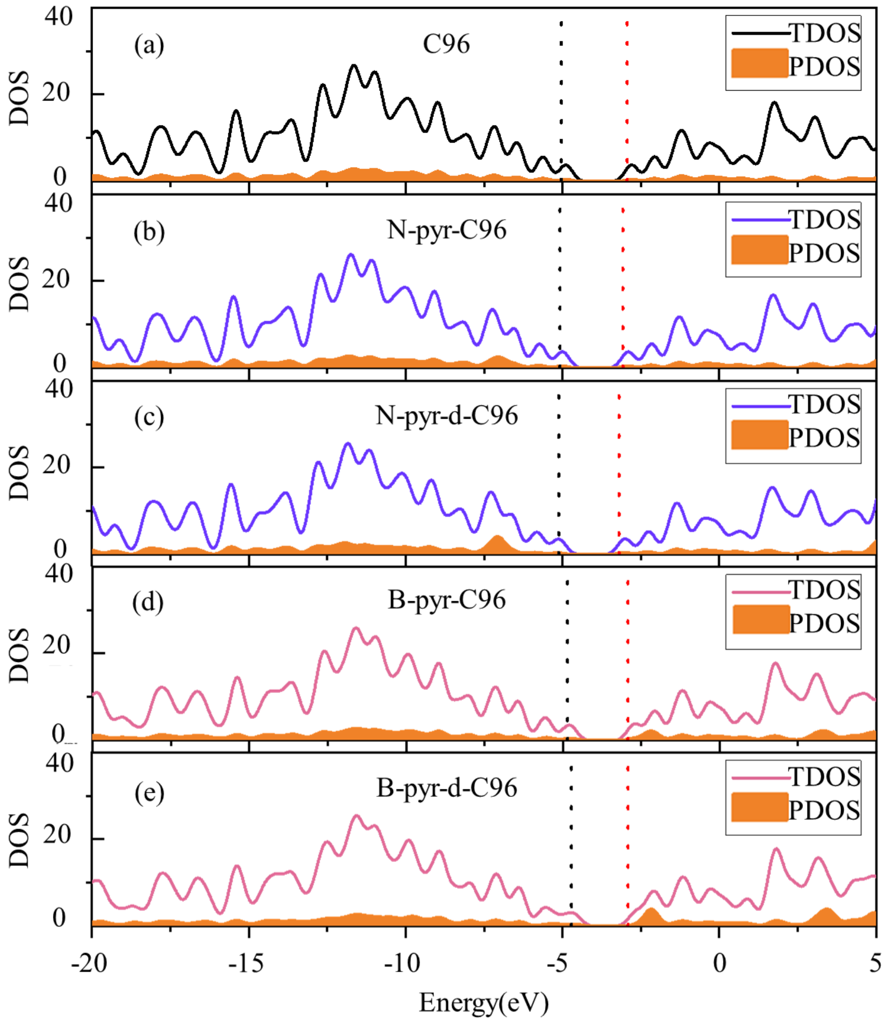

Fig. S2 DOS of C96 and Type-II-b defective C96. The solid line represents the TDOS. The yellow shaded regions represent the PDOS for the two benzene rings in C96, as well as the rings containing all defects in the defective C96. (The black dashed line represents the position of HOMO, the red dashed line represents the position of LUMO, and the DOS is plotted with an electron volt width of 0.5 eV.)

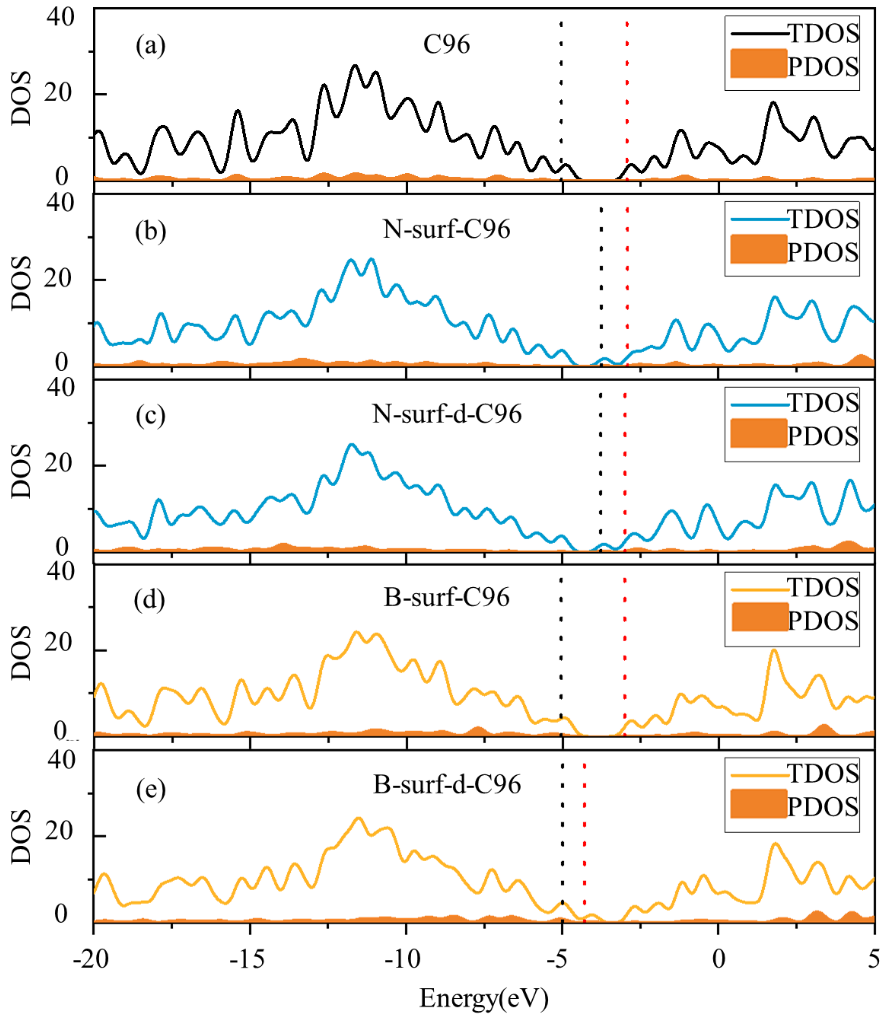

Fig. S3 DOS for C96 and Type-II-c defective C96. The solid line represents the TDOS. The yellow shaded regions represent the PDOS for the benzene ring in C96, as well as the rings containing all defects in the defective C96. (The black dashed line represents the position of HOMO, the red dashed line represents the position of LUMO, and the DOS is plotted with an electron volt width of 0.5 eV.)

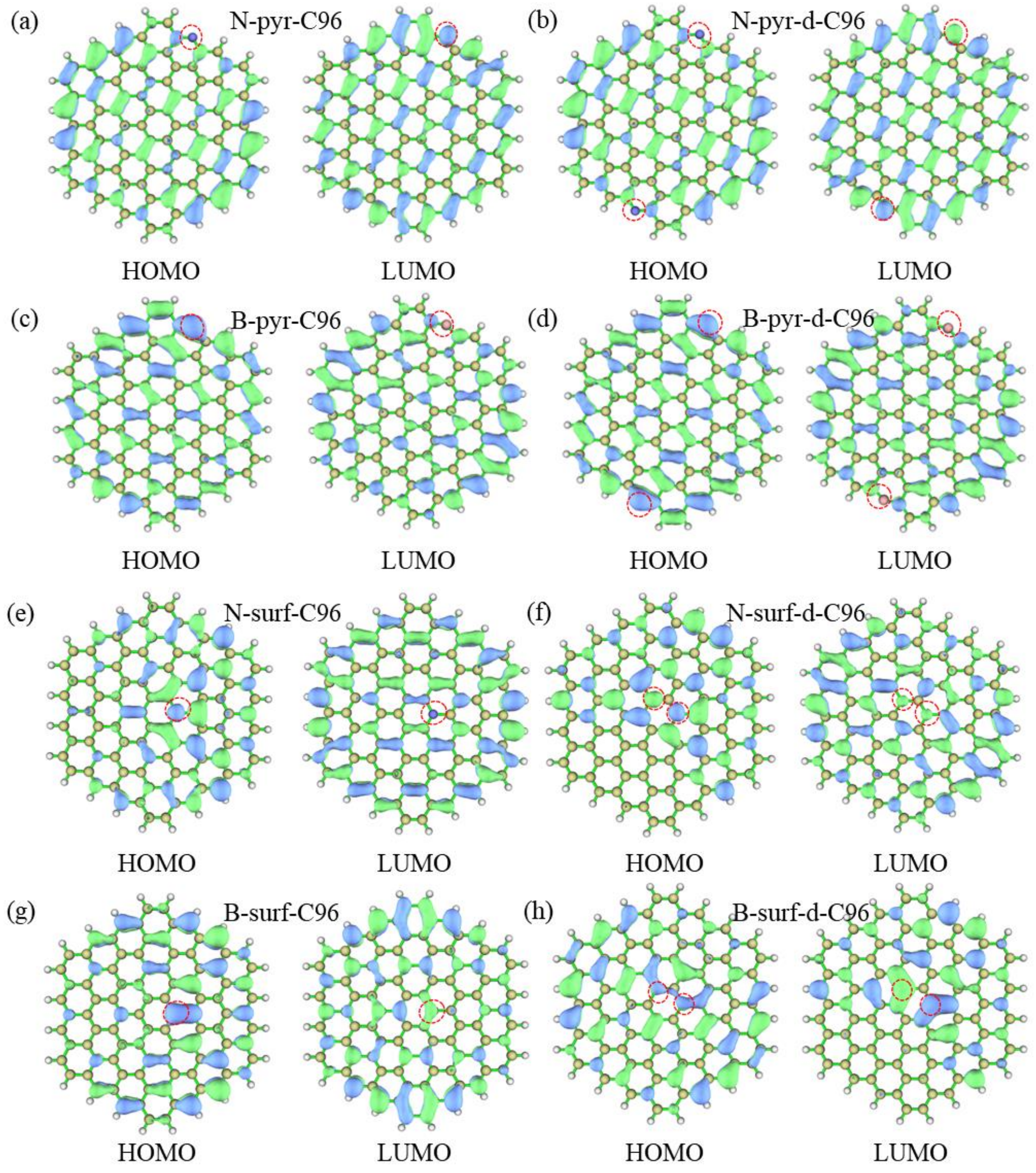

Fig. S4 The isosurfaces of HOMO and LUMO for a)-d) Type-II-b defective C96, e)-h) Type-II-c defective C96.

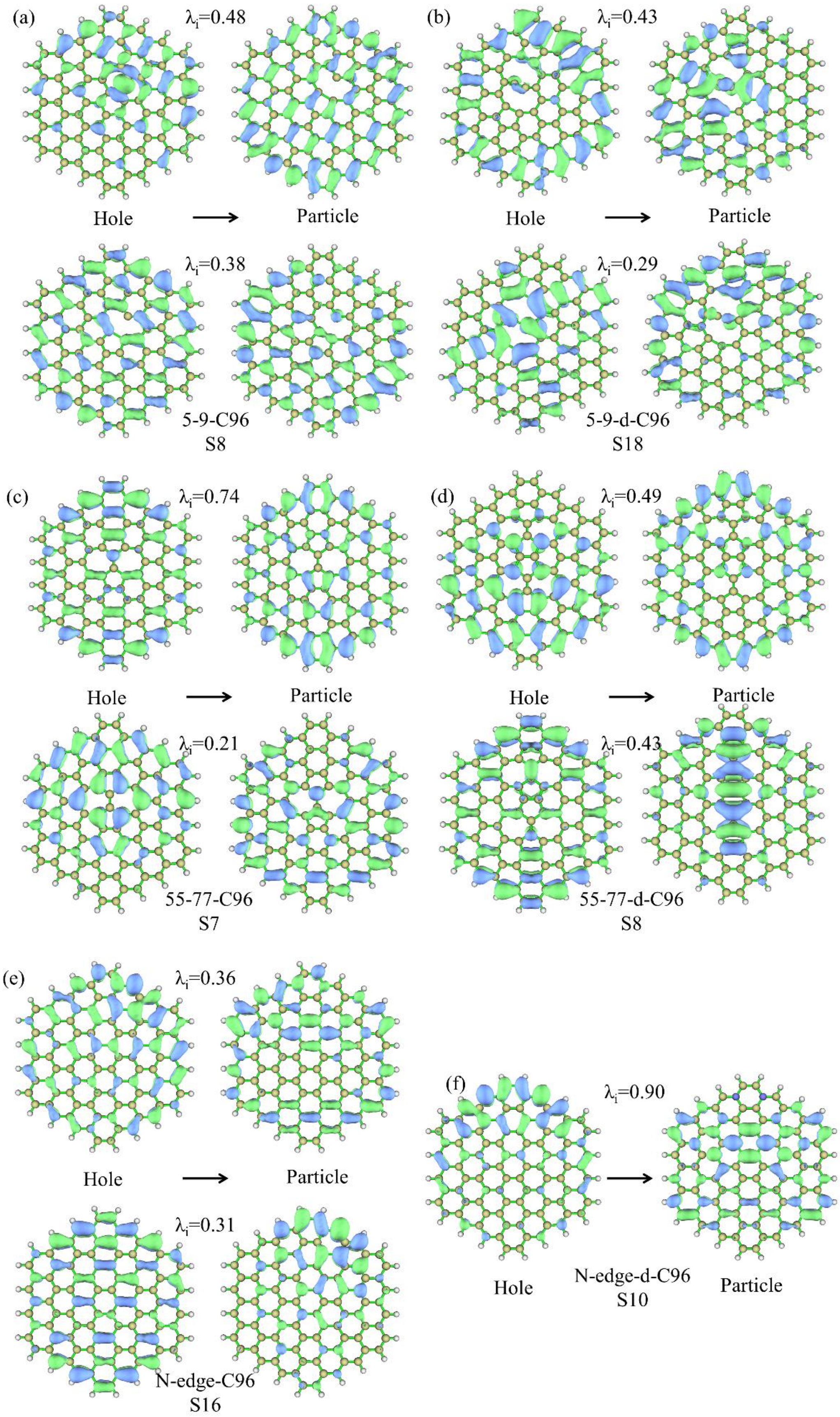

(a)
λi=0.48
Hole
Particle
λi=0.38
5-9-C96
S8
(b)
λi=0.43
Hole
Particle
λi=0.29
5-9-d-C96
S18
(c)
λi=0.74
Hole
Particle
λi=0.21
55-77-C96
S7
(d)
λi=0.49
Hole
Particle
λi=0.43
55-77-d-C96
S8
(e)
λi=0.36
Hole
Particle
λi=0.31
N-edge-C96
S16
(f)
λi=0.90
Hole
N-edge-d-C96
S10
Particle

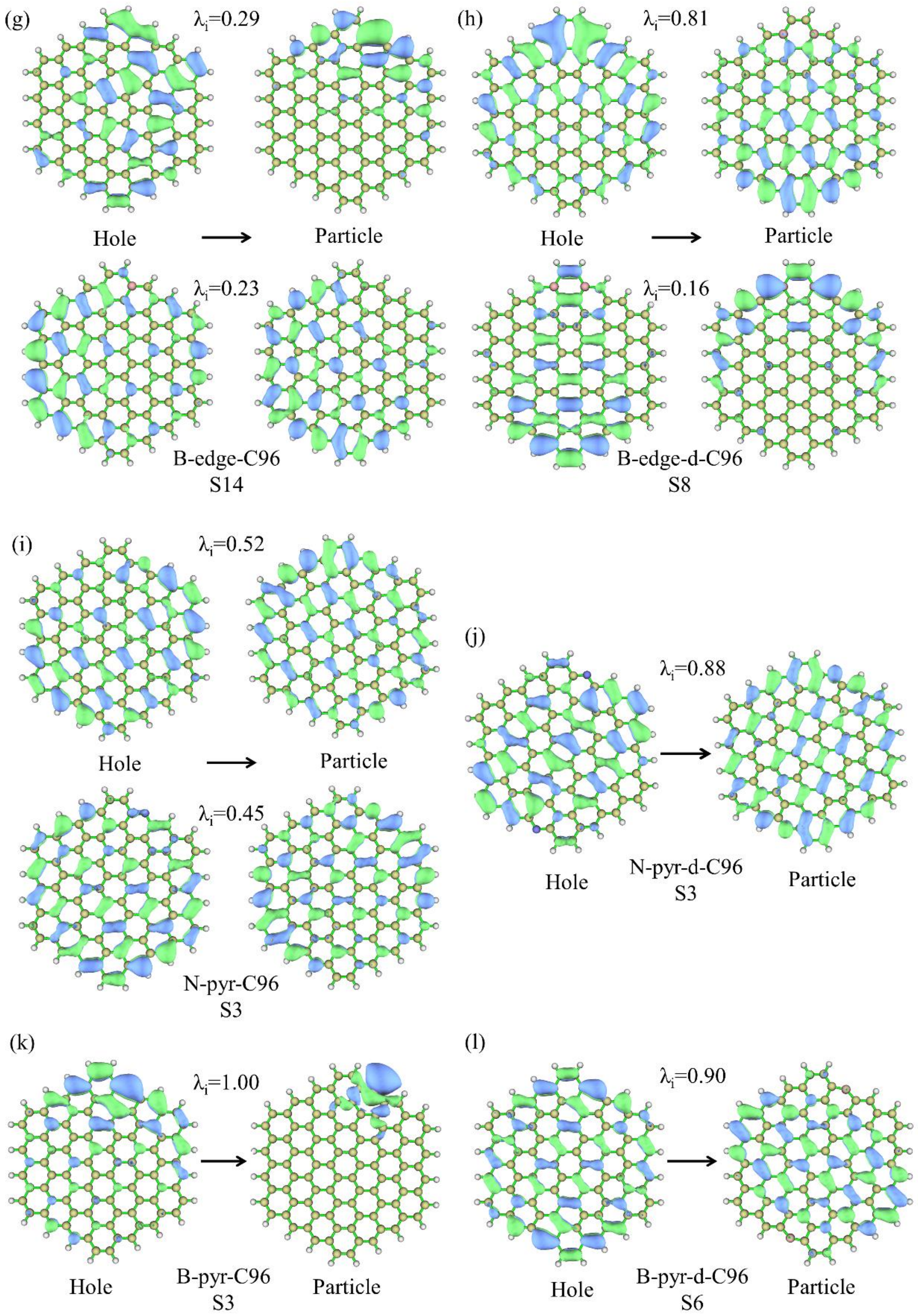
(g)
λi=0.29
Hole
Particle
λi=0.23
B-edge-C96
S14
(h)
λi=0.81
Hole
Particle
λi=0.16
B-edge-d-C96
S8
(i)
λi=0.52
Hole
Particle
λi=0.45
N-pyr-C96
S3
(j)
λi=0.88
Hole
N-pyr-d-C96
S3
Particle
(k)
λi=1.00
Hole
B-pyr-C96
S3
Particle
(l)
λi=0.90
Hole
B-pyr-d-C96
S6
Particle

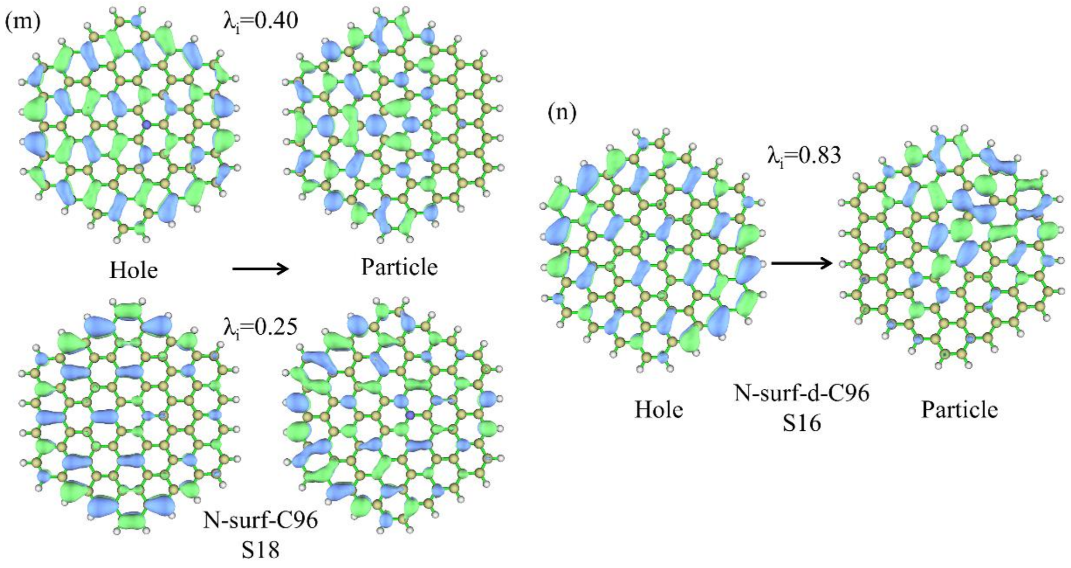

Fig. S5 NTO analysis for the prominent excited states of a)-d) Type-I defective C96, e)-n) Type-II defective C96. The hole is on the left and the particle is on the right; λ_i represents the corresponding eigenvalue.

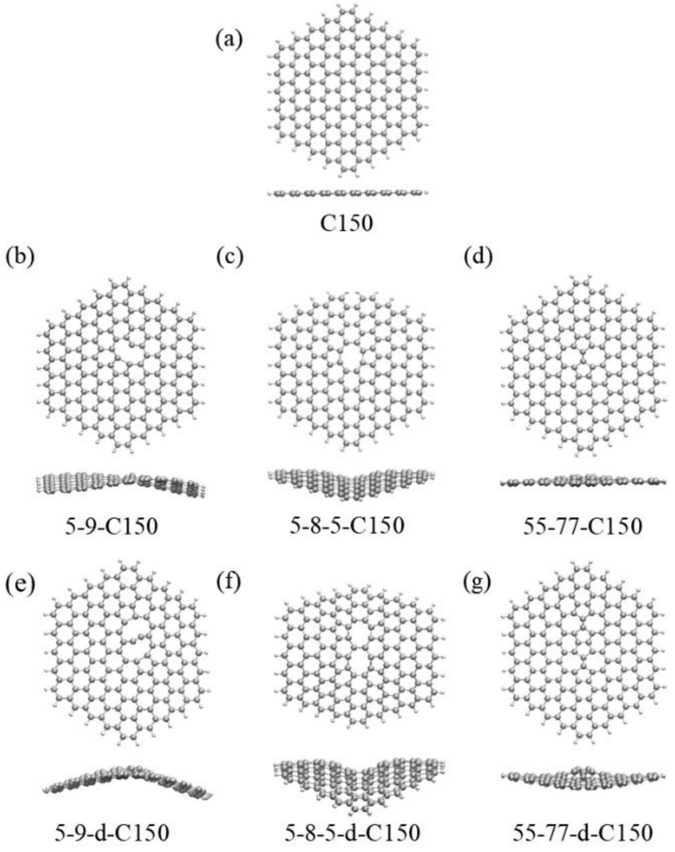

Fig.S6 The geometric structures of the optimized C150 and Type-I defective C150.

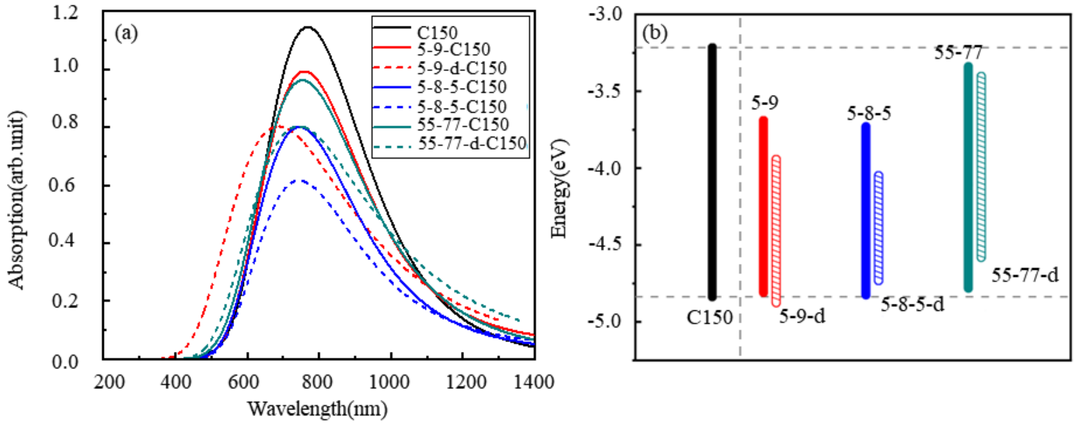

Fig. S7 (a) The calculated absorption spectra and (b) the HOMO-LUMO gaps of C150 and Type-I defective C150.

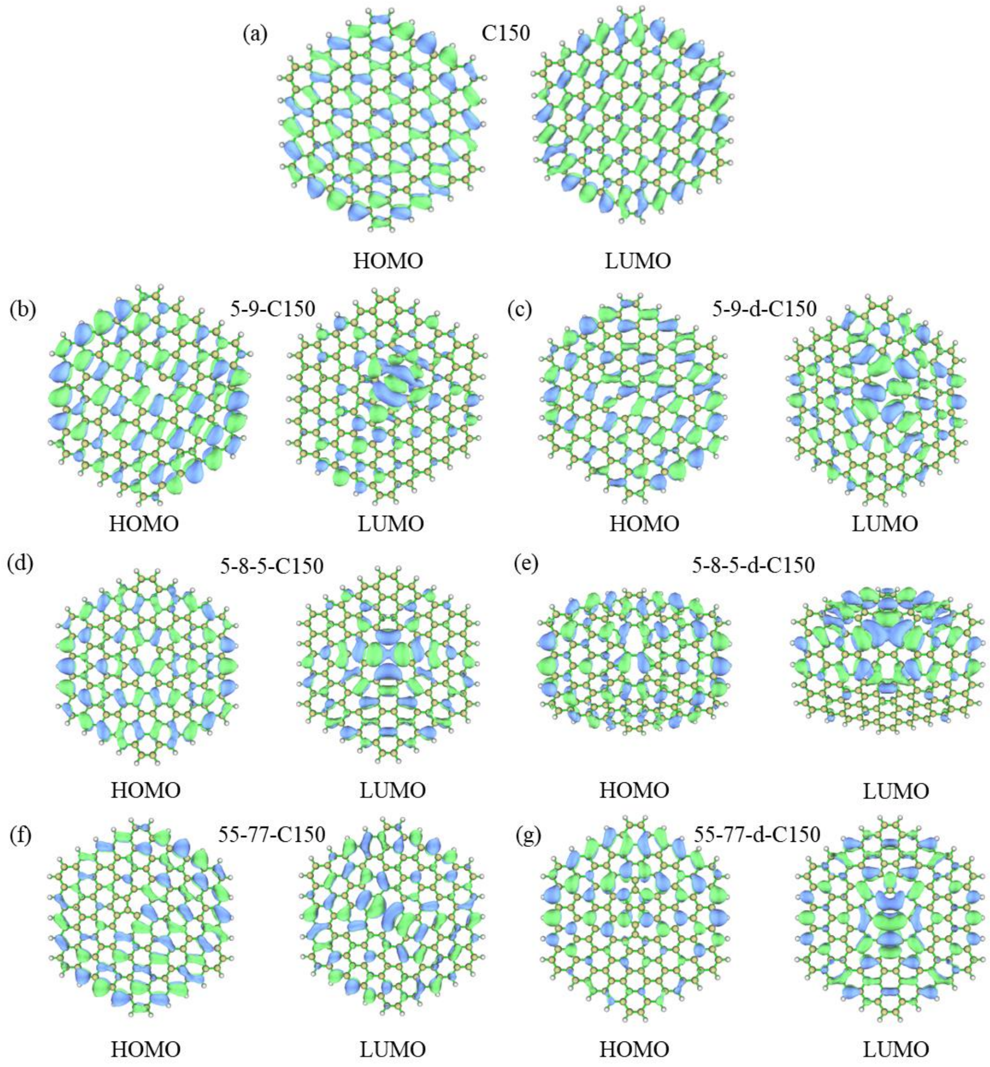

Fig. S8 The isosurfaces of HOMO and LUMO for a) C150, b)-g) Type-I defective C150.

Table S1 The HOMO energies, LUMO energies and HOMO-LUMO gaps for C96 and defective C96.

GQDs	HOMO(eV)	LUMO(eV)	gap(eV)
C96	-5.04	-2.94	2.10
5-9-C96	-5.04	-3.51	1.53
5-9-d-C96	-4.91	-3.99	0.92
5-8-5-C96	-4.97	-3.74	1.24
5-8-5-d-C96	-4.91	-3.82	1.09
55-77-C96	-4.86	-3.14	1.72
55-77-d-C96	-4.78	-3.24	1.54
N-edge-C96	-3.90	-2.94	0.96
N-edge-d-C96	-3.74	-3.03	0.71
B-edge-C96	-4.96	-2.96	2.00
B-edge-d-C96	-4.91	-4.28	0.62
N-pyr-C96	-5.08	-3.08	2.00
N-pyr-d-C96	-5.12	-3.20	1.92
B-pyr-C96	-4.85	-2.92	1.93
B-pyr-d-C96	-4.72	-2.91	1.81
N-surf-C96	-3.75	-2.91	0.84
N-surf-d-C96	-3.77	-2.99	0.78
B-surf-C96	-5.05	-3.01	2.04
B-surf-d-C96	-4.99	-4.29	0.71

Table S2 Absorption energies, wavelengths, and oscillator strengths for the first 20 singlet excited states of C96.

	Singlet states	Absorption energy (eV)	Wavelength (nm)	Oscillator strengths(f)
C96	1	1.65	749.45	0.0000
	2	1.79	694.50	0.0000
	3	2.09	592.48	1.7063
	4	2.09	592.54	1.7082
	5	2.34	529.84	0.0000
	6	2.34	529.84	0.0000
	7	2.35	527.95	0.0000
	8	2.42	511.68	0.0000
	9	2.42	511.41	0.0000
	10	2.47	502.11	0.0000
	11	2.47	502.09	0.0000
	12	2.52	492.37	0.0000
	13	2.52	492.27	0.0000
	14	2.67	464.91	0.0000
	15	2.67	464.84	0.0000
	16	2.70	458.40	0.0000
	17	2.98	416.74	0.0000
	18	2.99	415.03	0.0866
	19	2.99	414.97	0.0868
	20	3.00	413.43	0.0001

Table S3 Absorption energies, wavelengths, and oscillator strengths for the first 20 singlet excited states of 5-9-C96.

	Singlet states	Absorption energy (eV)	Wavelength (nm)	Oscillator strengths(f)
5-9-C96	1	1.04	1191.52	0.0179
	2	1.20	1032.14	0.0088
	3	1.63	759.73	0.0000
	4	1.72	721.99	0.0023
	5	1.75	708.62	0.0123
	6	1.83	677.65	0.0014
	7	1.85	670.87	0.0011
	8	2.10	591.05	1.4215
	9	2.11	587.10	1.2736
	10	2.14	578.24	0.0553
	11	2.30	538.84	0.0598
	12	2.33	533.08	0.0001
	13	2.33	531.79	0.0035
	14	2.37	522.07	0.0012
	15	2.40	516.82	0.0090
	16	2.44	508.95	0.0441
	17	2.53	489.75	0.0065
	18	2.54	488.08	0.0187
	19	2.55	485.37	0.0042
	20	2.60	477.12	0.0196

Table S4 Absorption energies, wavelengths, and oscillator strengths for the first 20 singlet states of 5-9-d-C96.

	Singlet states	Absorption energy (eV)	Wavelength (nm)	Oscillator strengths(f)
5-9-d-C96	1	0.47	2656.46	0.0206
	2	0.73	1692.15	0.0085
	3	1.17	1060.15	0.0519
	4	1.36	909.90	0.0358
	5	1.49	833.28	0.2370
	6	1.60	774.47	0.0365
	7	1.75	706.99	0.0223
	8	1.85	671.54	0.0083
	9	1.93	644.04	0.0013
	10	2.02	614.16	0.4463
	11	2.07	600.30	0.1286
	12	2.10	589.44	0.2149
	13	2.16	574.04	0.0233
	14	2.28	543.29	0.0062
	15	2.31	536.05	0.0200
	16	2.35	527.65	0.2279
	17	2.36	525.80	0.2857
	18	2.41	515.10	0.7125
	19	2.47	501.55	0.0342
	20	2.49	498.55	0.1396

Table S5 Absorption energies, wavelengths, and oscillator strengths for the first 20 singlet states of 5-8-5-C96.

	Singlet states	Absorption energy (eV)	Wavelength (nm)	Oscillator strengths(f)
5-8-5-C96	1	0.82	1506.01	0.0120
	2	0.89	1396.10	0.0724
	3	1.42	870.51	0.0019
	4	1.66	748.74	0.0186
	5	1.73	715.86	0.0354
	6	1.81	683.97	0.1419
	7	1.86	665.13	0.0547
	8	2.05	604.85	0.4053
	9	2.06	600.50	0.5264
	10	2.11	586.96	0.3584
	11	2.24	554.59	0.7693
	12	2.33	531.37	0.2176
	13	2.38	520.35	0.1735
	14	2.42	513.24	0.0000
	15	2.47	501.92	0.0581
	16	2.49	498.31	0.1914
	17	2.53	489.25	0.0020
	18	2.54	488.15	0.0040
	19	2.56	485.08	0.4194
	20	2.57	482.27	0.0113

Table S6 Absorption energies, wavelengths, and oscillator strengths for the first 20 singlet states of 5-8-5-d-C96.

	Singlet states	Absorption energy (eV)	Wavelength (nm)	Oscillator strengths(f)
5-8-5-d-C96	1	0.67	1859.88	0.0298
	2	1.12	1106.90	0.0138
	3	1.16	1064.62	0.0042
	4	1.28	968.63	0.0400
	5	1.48	835.73	0.0037
	6	1.64	754.51	0.0003
	7	1.72	721.48	0.0983
	8	1.74	713.69	0.0017
	9	1.75	710.51	0.0162
	10	1.81	685.57	0.0017
	11	1.90	654.21	0.1035
	12	1.95	636.36	0.0041
	13	2.04	606.94	0.4172
	14	2.04	606.85	0.6157
	15	2.05	604.21	0.0523
	16	2.19	564.99	0.0032
	17	2.23	555.37	0.0001
	18	2.33	531.74	0.0201
	19	2.35	526.94	0.0219
	20	2.38	519.91	0.0010

Table S7 Absorption energies, wavelengths, and oscillator strengths for the first 20 singlet states of 55-77-C96.

	Singlet states	Absorption energy (eV)	Wavelength (nm)	Oscillator strengths(f)
55-77-C96	1	1.30	954.70	0.0355
	2	1.51	819.42	0.0058
	3	1.68	738.45	0.1575
	4	1.73	714.76	0.2068
	5	1.95	637.01	0.0002
	6	2.00	618.71	0.9609
	7	2.04	606.35	1.1190
	8	2.21	561.27	0.0064
	9	2.21	560.76	0.0155
	10	2.28	543.39	0.3988
	11	2.30	539.84	0.0339
	12	2.33	532.05	0.0027
	13	2.39	518.61	0.0989
	14	2.46	505.03	0.0794
	15	2.46	504.39	0.0034
	16	2.55	485.96	0.0046
	17	2.56	483.86	0.0211
	18	2.57	482.13	0.0110
	19	2.59	478.39	0.1054
	20	2.60	475.99	0.0084

Table S8 Absorption energies, wavelengths, and oscillator strengths for the first 20 singlet states of 55-77-d-C96.

	Singlet states	Absorption energy (eV)	Wavelength (nm)	Oscillator strengths(f)
	1	1.10	1131.69	0.0015
	2	1.25	994.60	0.1243
	3	1.53	811.72	0.0408
	4	1.59	780.66	0.0002
	5	1.60	775.64	0.0371
	6	1.73	714.79	0.0003
	7	1.81	683.84	0.1747
	8	1.88	659.89	0.7068
	9	2.12	584.67	0.1924
	10	2.16	574.81	0.0228
55-77-d-C96	11	2.16	573.77	0.2524
	12	2.21	560.73	0.0317
	13	2.23	555.73	0.3942
	14	2.24	553.61	0.2637
	15	2.29	542.39	0.0913
	16	2.33	532.86	0.0166
	17	2.35	528.15	0.0221
	18	2.43	510.10	0.1671
	19	2.47	502.83	0.0131
	20	2.52	491.20	0.0123

Table S9 Absorption energies, wavelengths, and oscillator strengths for the first 20 singlet states of N-edge-C96.

	Singlet states	Absorption energy (eV)	Wavelength (nm)	Oscillator strengths(f)
N-edge-C96	1	0.48	2583.21	0.0101
	2	0.93	1329.66	0.1006
	3	1.15	1079.79	0.0057
	4	1.22	1018.29	0.0246
	5	1.29	958.81	0.0154
	6	1.54	805.71	0.0274
	7	1.60	774.68	0.0332
	8	1.64	754.32	0.0702
	9	1.69	732.40	0.0596
	10	1.73	715.09	0.0061
	11	1.76	704.70	0.0240
	12	1.84	674.76	0.0039
	13	1.88	660.91	0.0167
	14	1.92	646.20	0.0944
	15	1.99	623.25	0.6904
	16	2.03	611.40	0.7563
	17	2.05	604.93	0.0351
	18	2.10	589.16	0.1355
	19	2.12	585.89	0.0002
	20	2.16	573.42	0.3031

Table S10 Absorption energies, wavelengths, and oscillator strengths for the first 20 singlet states of N-edge-d-C96.

	Singlet states	Absorption energy (eV)	Wavelength (nm)	Oscillator strengths(f)
N-edge-d-C96	1	0.26	4813.79	0.0000
	2	0.89	1386.70	0.0906
	3	1.14	1086.91	0.0499
	4	1.28	972.32	0.2020
	5	1.30	956.04	0.2800
	6	1.54	805.91	0.0092
	7	1.81	686.83	0.0217
	8	1.81	685.43	0.0069
	9	1.88	658.96	0.5760
	10	1.90	652.01	0.8178
	11	2.01	616.98	0.0036
	12	2.08	596.08	0.0007
	13	2.09	594.46	0.0001
	14	2.16	575.29	0.0024
	15	2.27	546.28	0.5605
	16	2.28	543.34	0.1014
	17	2.35	527.72	0.6466
	18	2.38	521.93	0.0139
	19	2.40	517.09	0.0113
	20	2.46	503.21	0.0457

Table S11 Absorption energies, wavelengths, and oscillator strengths for the first 20 singlet states of B-edge-C96.

	Singlet states	Absorption energy (eV)	Wavelength (nm)	Oscillator strengths(f)
B-edge-C96	1	0.50	2504.89	0.0116
	2	0.96	1295.34	0.1263
	3	1.19	1042.22	0.0034
	4	1.23	1006.85	0.0154
	5	1.33	928.80	0.0136
	6	1.55	801.46	0.0231
	7	1.60	775.25	0.0530
	8	1.65	751.21	0.0723
	9	1.70	727.52	0.0379
	10	1.76	704.84	0.0170
	11	1.86	667.32	0.0250
	12	1.91	649.36	0.0667
	13	1.98	625.46	0.4400
	14	2.00	619.95	0.4770
	15	2.04	607.15	0.4523
	16	2.09	593.68	0.0984
	17	2.14	578.94	0.3382
	18	2.16	574.59	0.0075
	19	2.18	568.57	0.3883
	20	2.28	543.69	0.0886

Table S12 Absorption energies, wavelengths, and oscillator strengths for the first 20 singlet states of B-edge-d-C96.

	Singlet states	Absorption energy (eV)	Wavelength (nm)	Oscillator strengths(f)
B-edge-d-C96	1	0.18	6732.25	0.0002
	2	0.95	1300.61	0.1191
	3	1.15	1079.55	0.1758
	4	1.20	1037.54	0.0736
	5	1.22	1016.17	0.2870
	6	1.68	739.31	0.0187
	7	1.85	669.32	0.6529
	8	1.86	668.16	0.8075
	9	1.96	631.08	0.0454
	10	1.99	622.71	0.0100
	11	2.03	609.51	0.0101
	12	2.22	559.15	0.0255
	13	2.27	547.03	0.1769
	14	2.29	541.00	0.0003
	15	2.33	532.94	0.0000
	16	2.34	530.49	0.1779
	17	2.34	529.93	0.0335
	18	2.41	514.67	0.2915
	19	2.42	512.01	0.1046
	20	2.44	508.08	0.4587

Table S13 Absorption energies, wavelengths, and oscillator strengths for the first 20 singlet states of N-pyr-C96.

	Singlet states	Absorption energy (eV)	Wavelength (nm)	Oscillator strengths(f)
N-pyr-C96	1	1.64	755.83	0.0606
	2	1.77	698.77	0.0006
	3	2.07	598.47	1.5247
	4	2.08	594.83	1.5076
	5	2.30	538.18	0.0161
	6	2.33	532.21	0.0225
	7	2.38	521.86	0.0763
	8	2.40	515.97	0.0025
	9	2.41	513.45	0.0340
	10	2.47	502.18	0.0334
	11	2.48	499.08	0.0012
	12	2.54	487.95	0.0451
	13	2.55	486.35	0.0473
	14	2.67	464.16	0.0010
	15	2.68	463.21	0.0067
	16	2.71	457.67	0.0048
	17	2.98	416.00	0.0226
	18	3.00	413.86	0.0258
	19	3.02	410.35	0.0661
	20	3.06	405.74	0.0008

Table S14 Absorption energies, wavelengths, and oscillator strengths for the first 20 singlet states of N-pyr-d-C96.

	Singlet states	Absorption energy (eV)	Wavelength (nm)	Oscillator strengths(f)
N-pyr-d-C96	1	1.62	767.35	0.1981
	2	1.79	691.91	0.0044
	3	2.10	591.12	1.6076
	4	2.15	575.85	1.4627
	5	2.23	555.80	0.0000
	6	2.28	544.71	0.0000
	7	2.30	537.92	0.0000
	8	2.39	518.18	0.0000
	9	2.44	507.38	0.0000
	10	2.47	501.11	0.0000
	11	2.49	498.13	0.0000
	12	2.55	485.72	0.0000
	13	2.61	475.51	0.0000
	14	2.73	454.42	0.0000
	15	2.74	453.22	0.0000
	16	2.79	444.89	0.0000
	17	2.94	421.82	0.1753
	18	2.95	420.63	0.0126
	19	2.98	416.16	0.0148
	20	3.03	408.66	0.0597

Table S15 Absorption energies, wavelengths, and oscillator strengths for the first 20 singlet states of B-pyr-C96.

	Singlet states	Absorption energy (eV)	Wavelength (nm)	Oscillator strengths(f)
B-pyr-C96	1	1.61	771.67	0.1222
	2	1.74	711.55	0.0219
	3	2.04	607.90	1.3370
	4	2.06	602.06	0.0011
	5	2.07	600.27	1.3056
	6	2.19	565.58	0.1743
	7	2.24	552.38	0.0196
	8	2.34	528.79	0.1311
	9	2.37	523.68	0.0553
	10	2.42	512.25	0.0343
	11	2.48	500.37	0.0756
	12	2.49	498.84	0.0615
	13	2.50	495.66	0.0109
	14	2.56	483.76	0.0000
	15	2.57	483.34	0.0090
	16	2.63	472.09	0.0015
	17	2.70	459.23	0.0008
	18	2.72	455.89	0.0054
	19	2.82	439.51	0.0684
	20	2.84	436.90	0.0489

Table S16 Absorption energies, wavelengths, and oscillator strengths for the first 20 singlet states of B-pyr-d-C96.

	Singlet states	Absorption energy (eV)	Wavelength (nm)	Oscillator strengths(f)
B-pyr-d-C96	1	1.54	802.69	0.3094
	2	1.75	708.85	0.0171
	3	2.01	616.04	0.0000
	4	2.01	616.03	0.0021
	5	2.04	607.27	0.0000
	6	2.06	601.28	1.5939
	7	2.14	578.70	0.0000
	8	2.15	575.62	1.2346
	9	2.19	566.92	0.0000
	10	2.35	527.77	0.0000
	11	2.39	518.45	0.0000
	12	2.44	508.05	0.0000
	13	2.48	500.60	0.0000
	14	2.50	495.22	0.0000
	15	2.57	482.94	0.0000
	16	2.57	482.94	0.0000
	17	2.59	477.91	0.0000
	18	2.68	463.33	0.2630
	19	2.68	462.80	0.0065
	20	2.73	454.04	0.0000

Table S17 Absorption energies, wavelengths, and oscillator strengths for the first 20 singlet states of N-surf-C96.

	Singlet states	Absorption energy (eV)	Wavelength (nm)	Oscillator strengths(f)
N-surf-C96	1	0.33	3780.00	0.0032
	2	0.58	2153.35	0.0055
	3	0.90	1373.85	0.0172
	4	1.08	1143.53	0.0951
	5	1.21	1023.25	0.0064
	6	1.29	957.67	0.0121
	7	1.54	806.06	0.0004
	8	1.57	789.00	0.0096
	9	1.58	783.12	0.0273
	10	1.64	756.38	0.0049
	11	1.67	744.40	0.0541
	12	1.67	744.37	0.0389
	13	1.69	734.93	0.0240
	14	1.72	721.94	0.0256
	15	1.80	687.39	0.0084
	16	1.86	667.30	0.0001
	17	1.90	651.63	0.0239
	18	1.91	650.40	0.1643
	19	1.93	642.15	0.0002
	20	1.98	626.17	0.1304

Table S18 Absorption energies, wavelengths, and oscillator strengths for the first 20 singlet states of N-surf-d-C96.

	Singlet states	Absorption energy (eV)	Wavelength (nm)	Oscillator strengths(f)
N-surf-d-C96	1	0.40	3096.30	0.0275
	2	0.64	1923.19	0.0066
	3	0.65	1901.21	0.0109
	4	1.36	912.25	0.1476
	5	1.49	830.46	0.0088
	6	1.51	822.57	0.0641
	7	1.59	778.94	0.0051
	8	1.71	727.09	0.0254
	9	1.71	724.95	0.0007
	10	1.79	693.81	0.1104
	11	1.84	675.23	0.0275
	12	1.90	652.55	0.2635
	13	1.96	632.38	0.0525
	14	2.04	606.78	0.2170
	15	2.16	573.41	0.1432
	16	2.16	572.71	1.1002
	17	2.19	565.72	0.0327
	18	2.26	549.65	0.9313
	19	2.41	514.75	0.0737
	20	2.56	483.46	0.0558

Table S19 Absorption energies, wavelengths, and oscillator strengths for the first 20 singlet states of B-surf-C96.

	Singlet states	Absorption energy (eV)	Wavelength (nm)	Oscillator strengths(f)
B-surf-C96	1	0.30	4145.30	0.0029
	2	0.61	2028.83	0.0102
	3	0.93	1331.85	0.0788
	4	0.98	1259.46	0.0652
	5	1.25	991.14	0.0182
	6	1.39	891.41	0.0019
	7	1.57	787.99	0.0442
	8	1.59	777.68	0.0397
	9	1.63	762.16	0.0061
	10	1.67	741.72	0.0578
	11	1.70	727.28	0.0103
	12	1.77	699.73	0.0106
	13	1.80	689.35	0.0005
	14	1.82	681.79	0.0125
	15	1.93	640.95	0.0275
	16	1.94	639.47	0.2788
	17	1.97	628.22	0.0046
	18	1.99	621.74	0.1628
	19	2.01	615.93	0.6164
	20	2.02	612.69	0.0093

Table S20 Absorption energies, wavelengths, and oscillator strengths for the first 20 singlet states of B-surf-d-C96.

	Singlet states	Absorption energy (eV)	Wavelength (nm)	Oscillator strengths(f)
B-surf-d-C96	1	0.34	3674.90	0.0222
	2	0.60	2056.94	0.0007
	3	0.63	1976.90	0.0184
	4	1.24	1001.22	0.2372
	5	1.60	776.68	0.0165
	6	1.62	767.00	0.0410
	7	1.71	725.27	0.0078
	8	1.79	693.90	0.0899
	9	1.87	661.59	0.0706
	10	1.90	652.81	0.2626
	11	1.92	646.34	0.1013
	12	1.94	638.76	0.0027
	13	2.01	616.53	0.0001
	14	2.04	607.07	0.0260
	15	2.06	603.30	0.2697
	16	2.19	565.59	0.9884
	17	2.20	563.77	0.8985
	18	2.21	560.96	0.0025
	19	2.41	515.02	0.1365
	20	2.44	509.08	0.0628
